# Introducing the Australian Child Maltreatment Study: baseline evidence for a national public health challenge

**DOI:** 10.5694/mja2.51867

**Published:** 2023-04-02

**Authors:** James G Scott, Ben Mathews

**Affiliations:** ^1^ Child Health Research Centre, the University of Queensland Brisbane QLD; ^2^ QIMR Berghofer Medical Research Institute Brisbane QLD; ^3^ Queensland University of Technology Brisbane QLD; ^4^ Bloomberg School of Public Health Johns Hopkins University Baltimore MD United States of America

**Keywords:** Child welfare, Child abuse, Child health, Mental disorders

Public health initiatives are needed urgently to prevent the maltreatment of Australian children

Child maltreatment, comprising physical abuse, sexual abuse, emotional abuse, neglect, and exposure to domestic violence, is a serious public health concern. Its causal associations with health risk behaviours, physical illness and mental health problems are well established.^1^, ^2^ Despite the enormous burden of disease attributable to child maltreatment in Australia,^3^ there are significant gaps in our knowledge. Information is lacking on the prevalence of child maltreatment, trends over time, patterns of exposure to multiple forms of maltreatment, and associated health risk behaviours and health outcomes through life.

This supplement introduces the Australian Child Maltreatment Study (ACMS), the first national survey in the world to study in detail the experiences and associated health and social outcomes of all five forms of child maltreatment. Funded by the National Health and Medical Research Council, our multidisciplinary research team led by Professor Ben Mathews surveyed 8503 Australians aged 16 years and over, to ascertain detailed information on their experiences of child maltreatment.

Key findings from this landmark study are presented in six articles. Haslam and colleagues^4^ describe the study methods. A shortcoming of previous child maltreatment research has been the non‐standardised and unreliable way in which experiences are assessed. In the ACMS, child maltreatment was assessed using the Juvenile Victimisation Questionnaire‐R2 Adapted Version (Australian Child Maltreatment Study). To accurately assess experiences of child maltreatment, comprehensive methods were used to adapt the original Juvenile Victimisation Questionnaire^5^ to precisely capture a broad range of maltreatment experiences. Rigorous protocols were established to support the safety and welfare of participants and interviewers.^4^ The sample was representative of the national population and included an oversample of 3500 Australians aged 16–24 years.

Mathews and colleagues^6^ report the prevalence of each of the five forms of child maltreatment: physical abuse, 32.0% (95% CI, 30.7–33.3%); sexual abuse, 28.5% (95% CI, 27.3– 29.8%); emotional abuse, 30.9% (95% CI, 29.7–32.2%); neglect, 8.9% (95% CI, 8.1–9.7%); and exposure to domestic violence, 39.6% (95% CI, 38.3–40.9%). Women and gender diverse individuals experienced particularly high rates of child maltreatment. Comparison of prevalence of different forms of child maltreatment across age groups showed reductions in younger Australians for physical abuse and some types of sexual abuse, which coincides with increased awareness of the harm these forms of maltreatment cause to children. Of concern, most participants who experienced child maltreatment reported that it occurred many times or over many years.

Higgins and colleagues^7^ report the remarkable headline finding that the majority (62.2%) of Australians have experienced maltreatment in childhood. Most experienced multi‐type maltreatment, defined as the exposure to two or more of the five child maltreatment types. The prevalence of single‐type maltreatment was 22.8% (95% CI, 21.7–24.0%), whereas 39.4% (95% CI, 38.1–40.7%) reported multi‐type maltreatment. Nearly one‐quarter of Australians experienced three to five types of maltreatment. Exposure to domestic violence was the most prevalent individual maltreatment type, and was present across the most frequent multi‐type maltreatment patterns.

Lawrence and colleagues^8^ examined the association between child maltreatment and health risk behaviours of binge drinking, cannabis dependence, smoking, obesity, self‐harm, and suicide attempts. All five types of child maltreatment were associated with increased rates of all health risk behaviours, and these behaviours commenced during adolescence. Controlling for all five types of child maltreatment simultaneously, sexual abuse and emotional abuse were associated with highest odds of health risk behaviours.

Scott and colleagues^9^ assessed ACMS participants for mental disorder diagnoses of lifetime major depressive disorder, and of current alcohol use disorder, generalised anxiety disorder, and post‐traumatic stress disorder. The prevalence of mental disorders in non‐maltreated participants was 21.6% (95% CI, 19.9–23.3%). This increased to 36.2% (95% CI, 33.5–38.9%) for those who experienced a single type of maltreatment, and 54.8% (95% CI, 52.6–56.9%) for participants who experienced multi‐type maltreatment. Associations between experiences of child maltreatment and mental disorders were strongest for sexual abuse, emotional abuse, and multi‐type maltreatment. Adjustment for childhood and current financial hardship, and current socio‐economic status, did not significantly attenuate the associations.

Pacella and colleagues^10^ examined the associations between child maltreatment and health service use. Over the previous 12 months, compared with non‐maltreated Australians, those who experienced child maltreatment were more likely to have had an overnight hospital admission, a mental health admission, and multiple visits to their general practitioner. Increased health service use was particularly high in those who experienced multi‐type maltreatment.

The ACMS provides the first accurate prevalence estimates of experiences of child maltreatment in Australia. It is concerning to consider that the majority of Australians are maltreated as children. Further, most of these maltreated children experience multi‐type maltreatment over a protracted period of time on multiple occasions.^6^ Unsurprisingly, increased risks of severe and persistent health problems and increased health service use are associated with child maltreatment. The ACMS provides stark and chilling evidence of the hardship many children endure across their lives as a result of the actions of those responsible for caring for them.

Child protection is everyone's responsibility. Historically it has been siloed from other services, frequently leading to the fragmented management of co‐occurring child protection, health and social problems in the community. Prevention of child maltreatment is critical to improving the health of the Australian community. Widespread implementation of evidence‐based interventions addressing family risk factors for child maltreatment such as parental conflict, mental illness and substance use is critical to the prevention of health risk behaviours^8^ and mental illness.^11^ The closing article in this supplement is a comprehensive call to action for public health policy to forge a new era in child protection, child and adolescent health, and family support as a moral imperative and a nation‐building necessity.^12^

Australia has led the world with public health initiatives such as the plain packaging of cigarettes, restrictions on smoking, and the mandatory wearing of seatbelts. With robust evidence of the enormous health and social harms attributable to child maltreatment, there is an urgent need for nationally coordinated public health initiatives to prevent the maltreatment of Australian children.

## Open access

Open access publishing facilitated by The University of Queensland, as part of the Wiley – The University of Queensland agreement via the Council of Australian University Librarians.

## Competing interests

No relevant disclosures.

## Provenance

Not commissioned; externally peer reviewed.

## References

[1] Carr A , Duff H , Craddock F . A systematic review of reviews of the outcome of noninstitutional child maltreatment. Trauma Violence Abuse 2020; 21: 828‐843. 30249161 10.1177/1524838018801334

[2] Norman RE , Byambaa M , De R , et al. The long‐term health consequences of child physical abuse, emotional abuse, and neglect: a systematic review and meta‐analysis. PLoS Med 2012; 9: e1001349. 23209385 10.1371/journal.pmed.1001349 PMC3507962

[3] Moore SE , Scott JG , Ferrari AJ , et al. Burden attributable to child maltreatment in Australia. Child Abuse Negl 2015; 48: 208‐220. 26056058 10.1016/j.chiabu.2015.05.006

[4] Haslam DM , Lawrence DM , Mathews B , et al. The Australian Child Maltreatment Study (ACMS), a national survey of the prevalence of child maltreatment and its correlates: methodology. Med J Aust 2023; 218 (6 Suppl): S5‐S12. 37004182 10.5694/mja2.51869 PMC10953333

[5] Finkelhor D , Hamby SL , Ormrod R , Turner H . The Juvenile Victimization Questionnaire: reliability, validity, and national norms. Child Abuse Negl 2005; 29: 383‐412. 15917079 10.1016/j.chiabu.2004.11.001

[6] Mathews B , Pacella R , Scott JG , et al. The prevalence of child maltreatment in Australia: findings from a national survey. Med J Aust 2023; 218 (6 Suppl): S13‐S18. 37004184 10.5694/mja2.51873 PMC10953347

[7] Higgins DJ , Mathews B , Pacella R , et al. The prevalence and nature of multi‐type child maltreatment in Australia. Med J Aust 2023; 218 (6 Suppl): S19‐S25. 37004183 10.5694/mja2.51868 PMC10952595

[8] Lawrence DM , Hunt A , Scott JG , et al. The association between child maltreatment and health risk behaviours and conditions throughout life in the Australian Child Maltreatment Study. Med J Aust 2023; 218 (6 Suppl): S34‐S39. 37004181 10.5694/mja2.51877 PMC10952518

[9] Scott JG , Malacova E , Mathews B , et al. The association between child maltreatment and mental disorders in the Australian Child Maltreatment Study. Med J Aust 2023; 218 (6 Suppl): S26‐S33. 37004186 10.5694/mja2.51870 PMC10952950

[10] Pacella R , Nation A , Mathews B , et al. Child maltreatment and health service use: findings of the Australian Child Maltreatment Study. Med J Aust 2023; 218 (6 Suppl): S40‐S46. 37004185 10.5694/mja2.51892 PMC10952869

[11] Scott JG , Thomas HJ , Erskine HE . Improving Australia's population mental health: an ounce of prevention is worth a pound of cure. Aust N Z J Psychiatry 2019; 53: 470‐471. 30704258 10.1177/0004867418814960

[12] Mathews B , Thomas HJ , Scott JG . A new era in child maltreatment prevention: call to action. Med J Aust 2023; 218 (6 Suppl): S47‐S51. 37004187 10.5694/mja2.51872 PMC10952631

